# Sex differences in trigeminal neuralgia: a focus on radiological and clinical characteristics

**DOI:** 10.1007/s10072-023-06923-5

**Published:** 2023-07-12

**Authors:** Gianfranco De Stefano, Daniel Litewczuk, Cristina Mollica, Giuseppe Di Pietro, Eleonora Galosi, Caterina Leone, Pietro Falco, Maria Giulia Tullo, Francesca Caramia, Andrea Truini, Giulia Di Stefano

**Affiliations:** 1 Department of Human Neuroscience, Sapienza University of Rome, Viale Università 30, 00185 Rome, Italy; 2 Department of Statistical Sciences, Sapienza University of Rome, Rome, Italy; 3 Department of Neuroscience, Imaging and Clinical Science, G. d’Annunzio University of Chieti-Pescara, Chieti, Italy

**Keywords:** Trigeminal neuralgia, Sex differences, Paroxysmal pain, Trigeminal root, Neurovascular conflict, Multi-hit model

## Abstract

**Background:**

It is well established that trigeminal neuralgia is more prevalent in females than in males. Neurovascular compression with morphological changes of the trigeminal root represents the most recognized etiological factor. However, other factors may play a role in the framework of a multi-hit model. The primary aim of this study was to investigate sex differences in radiological and clinical characteristics of trigeminal neuralgia to better understand the multifactorial origin of this peculiar neuropathic pain condition.

**Methods:**

In this cross-sectional study patients with a definite diagnosis of primary trigeminal neuralgia were consecutively enrolled. Each patient underwent 3T MRI with sequences dedicated to the study of neurovascular compression. Major morphological changes of the trigeminal root were quantitatively assessed. Clinical characteristics were systematically collected through a dedicated questionnaire. A logistic regression model was implemented to predict radiological and clinical characteristics based on sex.

**Results:**

A total of 114 patients with classical (87) or idiopathic trigeminal neuralgia (27) were enrolled. Female sex was predictive for idiopathic trigeminal neuralgia. Male sex was predictive, among the comorbidities and clinical characteristics, for hypertension, the involvement of the left side and the second trigeminal division, alone or with the ophthalmic division.

**Discussion:**

The preponderance of TN in the female sex and the association between idiopathic TN and the female sex suggest the role of additional etiological factors in the framework of a multi-hit model. The identification of clinical variables predicted by sex suggests the possibility that distinct phenotypes, with peculiar pathophysiological and therapeutic aspects, may occur in females and males.

**Supplementary Information:**

The online version contains supplementary material available at 10.1007/s10072-023-06923-5.

## Introduction

Trigeminal neuralgia (TN) is an exemplary neuropathic facial pain condition with peculiar characteristics and hallmark signs, including trigger factors and refractory periods [1]. Trigger zones and paroxysmal pain sensation may be dissociated, probably due to a phenomenon of cross-excitation between somatosensory afferents, and a refractory period of several seconds or minutes usually occurs between paroxysms [2]. According to the etiology, TN is classified as classical TN, related to a neurovascular compression producing morphological changes on the trigeminal root; secondary TN, related to a major neurological disease, and idiopathic, of unknown etiology [3, 4]. The pathophysiological mechanism triggering paroxysmal pain is ascribed to the focal demyelination of primary trigeminal afferents near the entry of the trigeminal root into the pons, making the axons hyperexcitable and increasing their susceptibility to ectopic excitation, ephaptic transmission, and high-frequency discharges [1, 5]. This peculiar mechanism explains the remarkable efficacy of sodium channel blockers as first-choice medical treatments [4]. Although neurovascular compression with relevant morphological changes of the trigeminal root represents the most recognized etiological factor of TN, identifying idiopathic TN suggests other possible pathophysiological mechanisms in the framework of a multi-hit model [1, 5].

The incidence of TN increases with age and is higher among the female than the male sex [6]. Although a small posterior fossa in the female sex has been proposed as a possible concurrent factor in idiopathic TN [7], the comprehensive role of sex-based factors remains open. Recognition of the evidence underlying sex differences in TN may provide new insight into etiological and pathophysiological mechanisms and guide the development of effective treatments, providing better therapeutic options for patients tailored to pathophysiology.

The primary aim of this study was to investigate sex differences in radiological and clinical characteristics in patients with classical and idiopathic TN.

## Methods

### Study protocol

In this cross-sectional study, we prospectively screened consecutive patients visiting the Center for Neuropathic Pain at Sapienza University from December 2015 to December 2022. The inclusion criterion was a definite diagnosis of classical or idiopathic TN following accepted criteria (Suppl. Figure) [8, 9]. We excluded patients with a secondary TN diagnosis, orofacial pain other than TN, cognitive disturbances, or communication barriers. The diagnosis of TN was independently confirmed by two clinicians. The number of patients in the inclusion period determined the sample size. Clinical and radiological information was prospectively acquired; the dedicated analysis focusing on sex differences was designed in a second step. Each patient filled in a dedicated questionnaire focused on demographics and clinical characteristics and underwent MRI acquired with a 3T magnet (Siemens Verio, Erlangen, Germany) equipped with a 12-channel head coil. The MRI scans included 3D constructive interference in steady state images (CISS–TR 1000 ms, TE 132 ms, FOV 200–200 mm, slice thickness 0.5 mm, slice number 56, matrix 384–384) and 3D time-of-flight magnetic resonance angiography scan (TOF–TR 22 ms, TE 3.60 ms, FOV 181–200 mm, slice thickness 0.5 mm, matrix 696–768) to disclose best the trigeminal root neurovascular compression and the morphological changes of the nerve [10]. The institutional review board of Policlinico Umberto I, Sapienza University of Rome, approved the research. The study was performed in accordance with the Declaration of Helsinki. Written informed consent was obtained from each participant.

### Clinical assessment

Clinical information was systematically gathered through a dedicated questionnaire. Quantitative and qualitative variables, including demographics, pain distribution, quality of pain, presence of remission periods, familial occurrence, association with autonomic symptoms, comorbidities, drug responsiveness, and tolerability were collected. Among the comorbidities, depression was reported under the following conditions: 1. the diagnosis by a medical doctor and 2. current assumption of drugs for depression.

### Neuroimaging analysis

Neuroimaging analysis was mostly performed using the acquired CISS sequences. In these images, a neurovascular contact was detected when a vessel was in relation to the trigeminal root with no bright cerebrospinal fluid visible between the two structures. The two most recognized morphological changes of the trigeminal nerve root, dislocation, and atrophy were then quantitatively assessed [11, 12]. Images were transferred to a dedicated workstation, and a three-planar workspace was reconstructed using the software MIPAV (http://mipav.cit.nih.gov/). Dislocation was measured on the transverse plane. Using a mouse-driven cursor, a line was drawn from where the nerve emerges from the Meckel’s cave to where it penetrates the pons. Dislocation was considered present when the trigeminal root significantly diverged from this line, and it was then measured as the maximal distance between this line and the trigeminal border. To simplify the further analysis, we divided patients with dislocation into two categories: moderate if it was less than 3 mm and severe if it was greater than 3 mm, as used in previous literature [11]. For assessment of atrophy, we delineated the trigeminal nerve root with a mouse-driven cursor; then the volume of the nerve was automatically calculated by 3D reconstruction software, as a summation of all the delineated voxels, for both nerves. Two neuroradiologists, blinded to all clinical data, independently calculated the volume of both trigeminal nerve roots in each participant. We considered the mean trigeminal nerve root volume between the two observers. Since it is known from previous reports that in healthy subjects, the 95% confidence intervals of nerve asymmetry are below 5% [13], we considered four categories for further analysis: no atrophy if nerve asymmetry was under 5%, mild atrophy if it was 5–10%, moderate atrophy if it was 10–20%, and severe atrophy if it was greater than 20%.

Classical TN was defined in the presence of neurovascular compression causing dislocation and/or moderate or severe atrophy of the trigeminal root. Conversely, idiopathic TN was defined in the absence of neurovascular compression or in the case of neurovascular compression producing minor morphological changes on the trigeminal nerve root-like flattening, indentation [12].

### Statistical analysis

Descriptive summaries of the demographic, clinical, and radiological variables were provided as percentages and mean ± SD, respectively, for categorical and numerical variables.

Sex differences were preliminarily explored with Student’s *t*-tests for the numerical variables and either the chi-squared or Fisher’s exact test for the categorical ones. As a confirmatory approach to the exploratory association analysis, we estimated a logistic regression model with sex as the binary outcome and the following variables as potential predictors: age at onset, side, division, etiology, phenotype (TN with purely paroxysmal pain and TN with concomitant continuous pain), remission periods, autonomic symptoms, familial occurrence, comorbidities including depression and hypertension, refractoriness to pharmacological treatment, adverse events to pharmacological treatment and drug discontinuation, presence of neurovascular compression, and morphological changes of the trigeminal root, including quantitative assessment of dislocation and atrophy. The use of logistic regression to predict socio-demographic characteristics such as sex and race is endorsed, for example, in those medical fields where there is interest in sex determination of unknown individuals from biological traits, as in legal medicine and forensic anthropology [14, 15]. The optimal set of variables for predicting sex was identified with the backward selection procedure [16] based on the minimization of the Akaike information criterion [17]. The overall performance of the resulting logistic regression model, as a tool for identifying sex-discriminating features in the TN condition, has been then assessed with the ROC and the quantification of the AUC.

All the tests performed in the analysis were two sided and a *p*-value < 0.05 was considered statistically significant. The statistical analysis was performed with the R software [18].

## Results

**Fig. 1:**
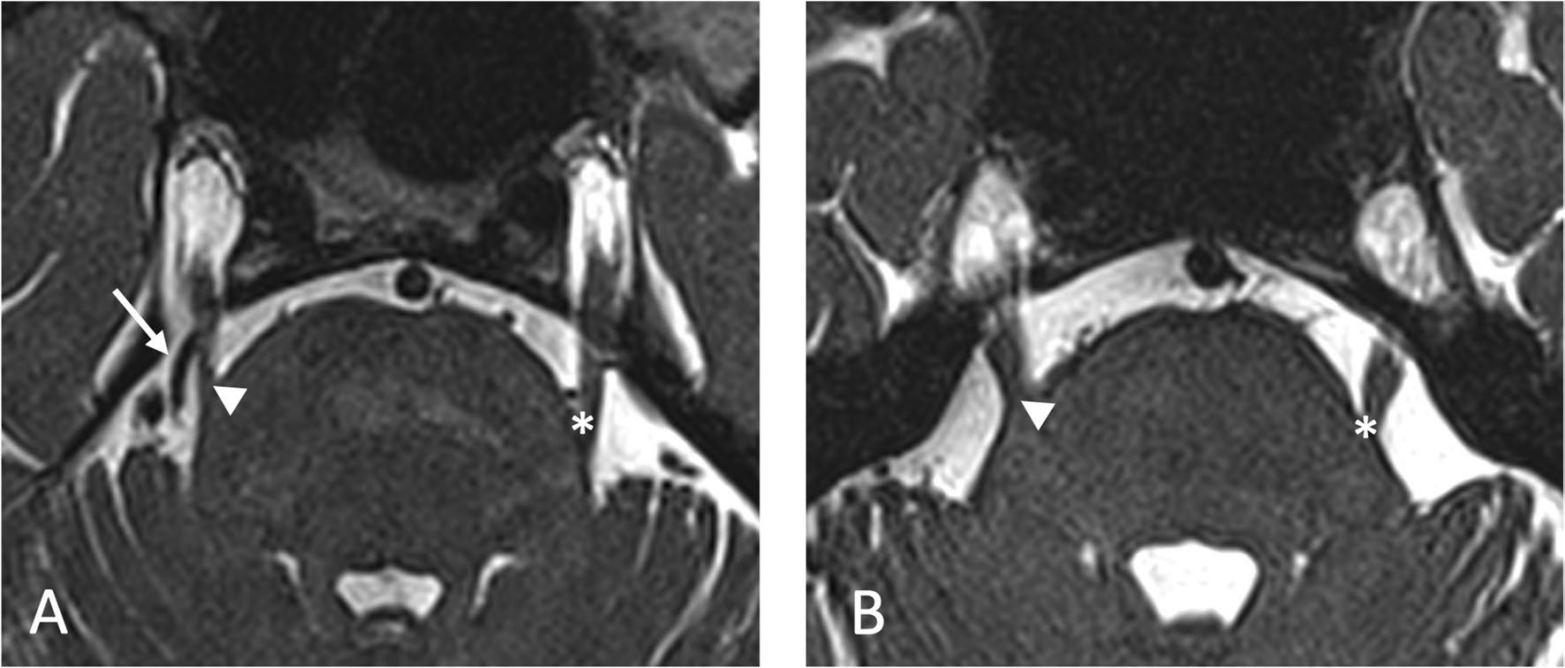
MRI CISS images of an exemplary patient with classical (**A**) and idiopathic (**B**) right TN. In (**A**) a vessel (arrow) impacts the right trigeminal root (arrowhead), which appears atrophic compared to the contralateral (star). In (**B**) no clear pathology affects the right trigeminal root (arrowhead), which appears similar to the contralateral (star)

**Fig. 2:**
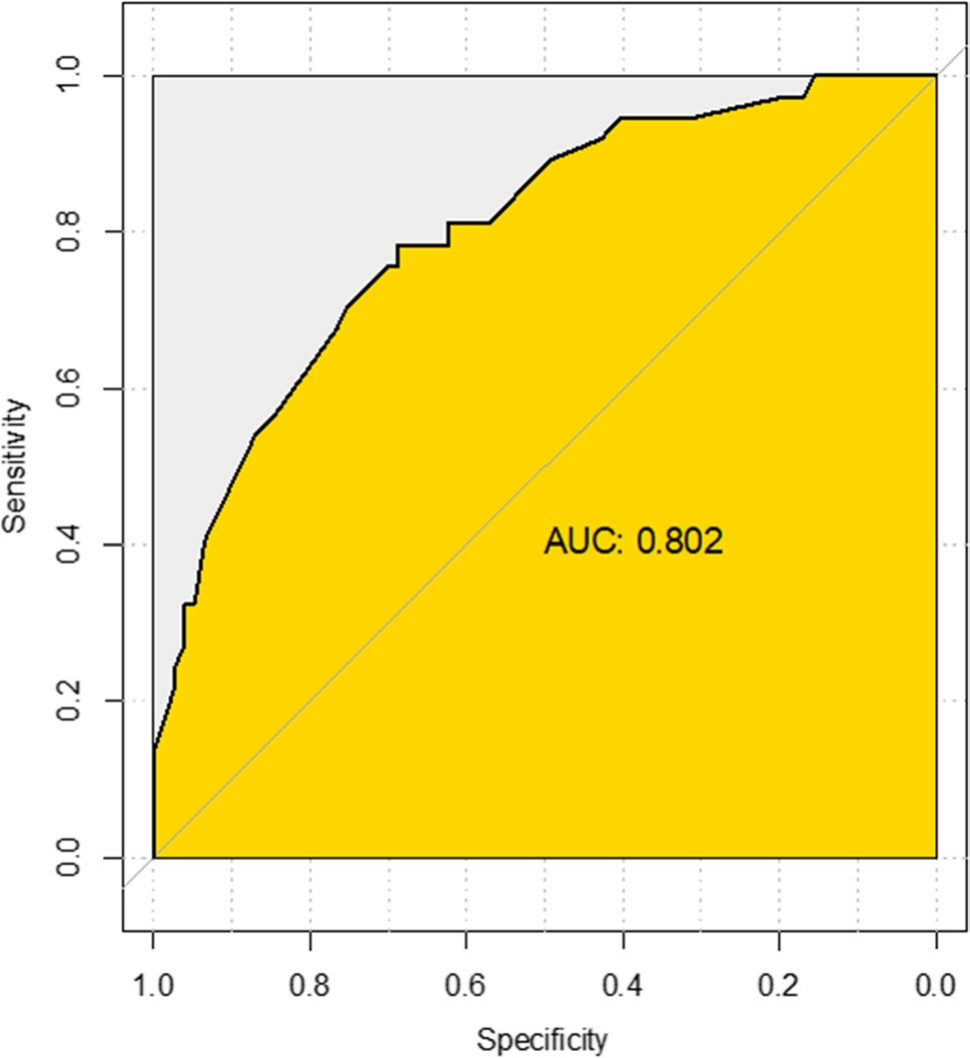
ROC curve and AUC of the logistic regression for sex prediction

We screened 145 patients with a definite diagnosis of TN and excluded 31 patients (18 patients with TN related to multiple sclerosis, five with a benign tumor of the posterior fossa, one with TN secondary to a superior cerebellar artery aneurysm, and seven with bilateral abnormal trigeminal reflex testing suggesting trigeminal neuropathy). We included in the analysis 114 patients with primary TN, 87 with classical, and 27 with idiopathic TN (77 females and 37 males, age 64.4 ± 13.2) (Fig. 1, Suppl. Figure). Demographics, clinical characteristics of TN, and radiological characteristics of the trigeminal nerve root in the female and in the male sex are summarized in Table 1. There were no missing data. The exploratory association analysis showed that classical TN significantly prevailed in the male sex (*p* = 0.033), as well as the involvement of the first trigeminal division (*p* = 0.034). Among the comorbidities, depression significantly prevailed in the female sex (*p* = 0.029). Adverse events due to pharmacological treatment with voltage-gated sodium channel blockers significantly prevailed in the female sex (*p* = 0.050) (Suppl. Table); consequently, drug discontinuation due to adverse events was more frequent in the female sex (*p* = 0.009). The optimal set of sex-related variables according to the backward selection procedure is summarized in Table 2. The ROC shows a very good accuracy performance of our multiple logistic regression approach in highlighting sex differences, corresponding to an AUC equal to 0.80 (Fig. 2). According to the final model, the female sex was predictive for idiopathic TN (*p* = 0.034). Male sex was predictive, among the clinical characteristics, for hypertension (*p* = 0.036), the involvement of the left side (*p* = 0.031), and the second trigeminal division alone (*p* = 0.006) or with the ophthalmic division (*p* = 0.038).

**Table 1:** Demographic, clinical, and anatomical characteristics by sex in patients with trigeminal neuralgia

Variable	Females *N* (%)	Males *N* (%)	*p*-value
Right sided	53 (68.8)	20 (54.1)	0.183
Left sided	24 (31.2)	17 (45.9)	
V1	2 (2.6)	3 (8.1)	**0.034**
V2	12 (15.6)	12 (32.4)	
V3	26 (33.8)	5 (13.5)	
V1–V2	11 (14.3)	9 (24.3)	
V2–V3	20 (26)	6 (16.2)	
V1–V2–V3	6 (7.8)	2 (5.4)	
Classical TN	54 (70.1)	33 (89.2)	**0.033**
Idiopathic TN	23 (29.9)	4 (10.8)	
TN with purely paroxysmal pain	39 (50.6)	22 (59.5)	0.495
TN with concomitant continuous pain	38 (49.4)	15 (40.5)	
With remission periods	53 (68.8)	23 (62.2)	0.621
Without remission periods	24 (31.2)	14 (37.8)	
With autonomic symptoms	18 (23.4)	9 (24.3)	1.000
Without autonomic symptoms	59 (76.6)	28 (75.7)	
With familial occurrence	8 (10.4)	3 (8.1)	1.000
Without familial occurrence	69 (89.6)	34 (81.9)	
With depression	10 (13)	0 (0)	**0.029**
Without depression	67 (87)	37 (100)	
With hypertension	26 (33.8)	19 (48.6)	0.111
Without hypertension	51 (66.2)	18 (51.4)	
Adverse events	33 (50)	10 (27.8)	**0.050**
Without adverse events	33 (50)	26 (72.2)	
Drop-out due to adverse events	18 (27.3)	2 (5.6)	**0.009**
Without drop-out due to adverse events	48 (72.7)	34 (94.4)	
No responders to pharmacological treatment	10 (13)	6 (16.2)	0.086
Responders to pharmacological treatment	67 (87)	31 (83.8)	
With neurovascular conflicts	63 (81.8)	35 (94.6)	0.086
Without neurovascular conflicts	14 (18.2)	2 (5.4)	
Multiple vessels with relationships to the root	31 (48.4)	19 (54.3)	0.729
Without multiple vessels with relationships to the root	33 (51.6)	16 (45.7)	
Atrophy of trigeminal nerve root	51 (66.2)	28 (75.7)	0.420
Without atrophy	26 (33.8)	9 (24.3)	
Absent/mild atrophy	30 (39)	13 (35.1)	0.765
Moderate atrophy	18 (23.4)	11 (29.7)	
Severe atrophy	29 (37.7)	13 (35.1)	
Dislocation of trigeminal nerve root	42 (63.6)	29 (80.6)	0.121
Without dislocation	24 (36.4)	7 (19.4)	
Absent dislocation	24 (36.4)	7 (20.6)	0.234
Less than 3 mm dislocation	29 (43.9)	17 (50)	
More than 3 mm dislocation	13 (19.7)	10 (29.4)	
Minor morphological changes	11 (14.3)	2 (5.4)	0.217
Without minor morphological changes	66 (85.7)	35 (94.6)	
Variable	Females mean ± SD	Males mean ± SD	*p*-value
Age at onset (years)	57.10 ± 12.3	58.62 ± 14.8	0.566

Descriptive summaries are expressed as frequencies (*N* and %) and mean ± SD for, respectively, categorical and numerical variables

For categorical variables: *p*-values refer to the chi-squared or exact Fisher’s test, as appropriate

For numerical variables: *p*-value refers to the *t*-test

*p*-values < 0.05 are marked in bold

**Table 2:** Results of the optimal logistic regression model obtained with the backward variable selection procedure

Variable	Odds ratio	95%CI	*p*-value
Left sided *(vs right sided)*	**2.99**	**[1.12,8.42]**	**0.031**
V1	7.26	[0.83,77.22]	0.076
V2	**7.42**	**[1.90,34.32]**	**0.006**
V1–V2	**4.60**	**[1.14,20.95]**	**0.038**
V2–V3	2.12	[0.48,9.81]	0.322
V1–V2–V3	2.79	[0.29,22.14]	0.334
Idiopathic etiology *(vs classic)*	**0.24**	**[0.06,0.83]**	**0.034**
With hypertension	**2.84**	**[1.09,7.84]**	**0.036**

*CI*, confidence interval

*p*-value refers to the two-sided tests of significance of the estimated adjusted odds ratios of the logistic regression models involving age at onset, side, division, etiology, phenotype, remission period, autonomic symptoms, familial occurrence, depression, hypertension, refractoriness to pharmacological treatment, morphological changes of trigeminal root as predictors of the gender

Odds ratios and confidence intervals have been computed by exponentiating the point and interval estimates of the regression coefficients. Odds ratio values are referred to the male sex

*p*-values < 0.05, the relevant odds ratio and 95%CI are marked in bold

## Discussion

In this clinical and neuroimaging study, a logistic regression model was applied to determine sex differences in patients diagnosed with TN in a multivariate framework which, differently from the more conventional bivariate analysis, allows to control for possible confounding factors. From the analysis of the radiological variables of the trigeminal nerve root, the female sex was predictive for idiopathic TN. Among the comorbidities and clinical characteristics, the male sex was predictive for hypertension, the involvement of the left side, and the second trigeminal division, alone or with the ophthalmic division. The application of ROC and the value of the AUC equal to 0.80 demonstrate a very good accuracy performance of the predictive variables found with our multiple logistic regression approach in highlighting sex differences. This evidence reinforces the impact of the clinical findings of our study, as possible expression of distinct phenotypes, and endorses their pathophysiological implications in the context of a multi-hit model.

Idiopathic TN was associated with the female sex. This finding is coherent with previous clinical and neuroimaging studies showing that females with TN are younger [19] and have a lower prevalence of severe neurovascular conflict on the symptomatic side [12]. Conversely, neurovascular compression was more frequently detected in the male sex, thus confirming a difference in the neuroanatomy in males and females with TN [20]. Additionally, the female sex was reported as a predictor of pain recurrence after microvascular decompression [21]. These data suggest that in the female sex, more frequently, there are etiological factors other than neurovascular compression contributing to TN. Although neurovascular compression with morphological changes of the trigeminal root represents the most recognitive etiological factor in TN, other factors increasing the vulnerability of primary trigeminal afferents in the female sex should be investigated in the context of a multi-hit model. Increasing evidence supports the role of rare variants of genes encoding voltage-gated ion channels and transient receptor potential channels [22–24]. Other factors related to neuroinflammation with arachnoiditis [25, 26], levels of gonadal hormones, and function of endogenous pain modulation have been supposed [27]. The recurrence of idiopathic TN in the female sex may tailor future research to these new etiological and pathophysiological factors.

TN in the male sex seems to be related to neurovascular compression, producing dislocation and/or atrophy on the trigeminal nerve root. In our cohort of patients, the male sex was predictive for hypertension. This finding is coherent with the hypothesis that arterial wall hardening due to high blood pressure may promote the development of morphological abnormalities in the nearby trigeminal root [28].

Male sex was predictive for the involvement of the second trigeminal division alone and in association with the ophthalmic division. The somatotopy of the trigeminal nerve root can explain this predominant involvement in the site of neurovascular conflict, more frequently detected at the supero-medial level [29, 30], thus reinforcing the etiologic role of neurovascular conflict in males.

In our exploratory analysis, the involvement of the first trigeminal division alone prevailed in the male sex. The ophthalmic division is the less frequently affected division in TN, probably due to the small number of myelinated fibers in V1 compared to V2 and V3 [30, 31]. The isolated involvement of the ophthalmic division may also represent a distinct phenotype and may recall the clinical picture of short-lasting unilateral neuralgiform headache attacks. A neuroimaging study has suggested the role of ipsilateral trigeminal neurovascular compression with morphological changes also in the pathophysiology of this disorder [32], and recent studies reported the efficacy of microvascular decompression [33] and sodium channel blockers [34], suggesting that this disease may share some pathophysiological mechanisms with TN.

We found that the male sex was predictive of the involvement of the left side. It is well known that TN affects more frequently the right side and the female sex. A historical study explains this right preponderance with an upward displacement of the apex of the petrous bone due to a basilar impression on the right side of postmenopausal females [35]. Some authors reported a narrower foramen rotundum and foramen ovale on the right side [36]; however, this interpretation is not coherent with the recognized pathophysiological mechanism of the focal demyelination involving the trigeminal root near the entry into the pons. Our clinical observations, including affected divisions and sides, raise the possibility that TN may occur with different phenotypes in the male and in the female sex. The pathophysiological mechanisms underlying these phenotypes deserve further analysis. Our neuroradiological analysis focused on morphological changes of the trigeminal root near the entry into the pons. Future neuroradiological studies of other possible anatomical differences among females and males are needed.

Although concomitant continuous pain was reported more frequently in females [37], in our study the female sex was not predictive for this clinical phenotype of TN. Recent studies reported trigeminal root atrophy with unmyelinated fiber dysfunction in patients with concomitant continuous pain, suggesting the association between this major morphological change in the site of neurovascular conflict and a central mechanism of denervation supersensitivity, triggered in second-order trigeminal neurons [13, 38].

The exploratory analysis showed poor tolerability of first-line drugs (carbamazepine and oxcarbazepine) in the female sex, with more drug discontinuation or dosage reduction to an unsatisfactory level. However, an impact of depression on subjective side effects cannot be ruled out. These findings, together with the significantly higher prevalence of depression, point out the burden of this excruciating condition in the female sex and the need for a tailored therapeutic approach [39], considering sex-specific clinical characteristics and vulnerabilities.

In the present study patient population was composed only of cis-gender people, who identified their gender the same as their biological sex, so no information can be drawn about clinical and radiological characteristics of TN in gender minorities. It has been shown that gender minority patients may have different phenotypes of numerous neurological diseases and may need different and personalized treatments [40]. Further studies, including more patients, are needed to disclose if TN in gender-minority patients has peculiar clinical or therapeutic characteristics.

## Conclusion

Although neurovascular compression represents the most recognized factor in TN, idiopathic TN was associated with the female sex. The preponderance of TN in females and the association between idiopathic TN and the female sex suggest the role of additional etiological factors in the framework of a multi-hit model. The identification of clinical variables predicted by the male sex suggests the possibility that distinct phenotypes, with peculiar pathophysiological and therapeutic aspects, may occur in the female and in the male sex. A complete understanding of the role of sex-based factors is crucial for a better comprehension of etiological and pathophysiological mechanisms and for reducing the burden of TN in females and males.

### Supplementary Information

ESM 2: hed_strobe_checklist (DOC 85 kb)

ESM 3: **Suppl. Figure.** Flow diagram of patients’ enrollment. (PNG 32 kb)

High resolution image (TIF 82 kb)

ESM 4: Suppl. Table. Adverse events related to carbamazepine or oxcarbazepine in 114 patients with Trigeminal Neuralgia (DOCX 13 kb)

## Data Availability

The datasets generated during and/or analyzed during the current study are available from the corresponding author on reasonable request.

## Abbreviations

TN: trigeminal neuralgia
CISS: constructive interference in steady state
