# Supplementary figures and images for: Sex differences in trigeminal neuralgia: a focus on radiological and clinical characteristics
Source: Neurol Sci. 2023 Jul 12;44(12):4465–72. doi: 10.1007/s10072-023-06923-5 (PMC10641090; doi:10.1007/s10072-023-06923-5)

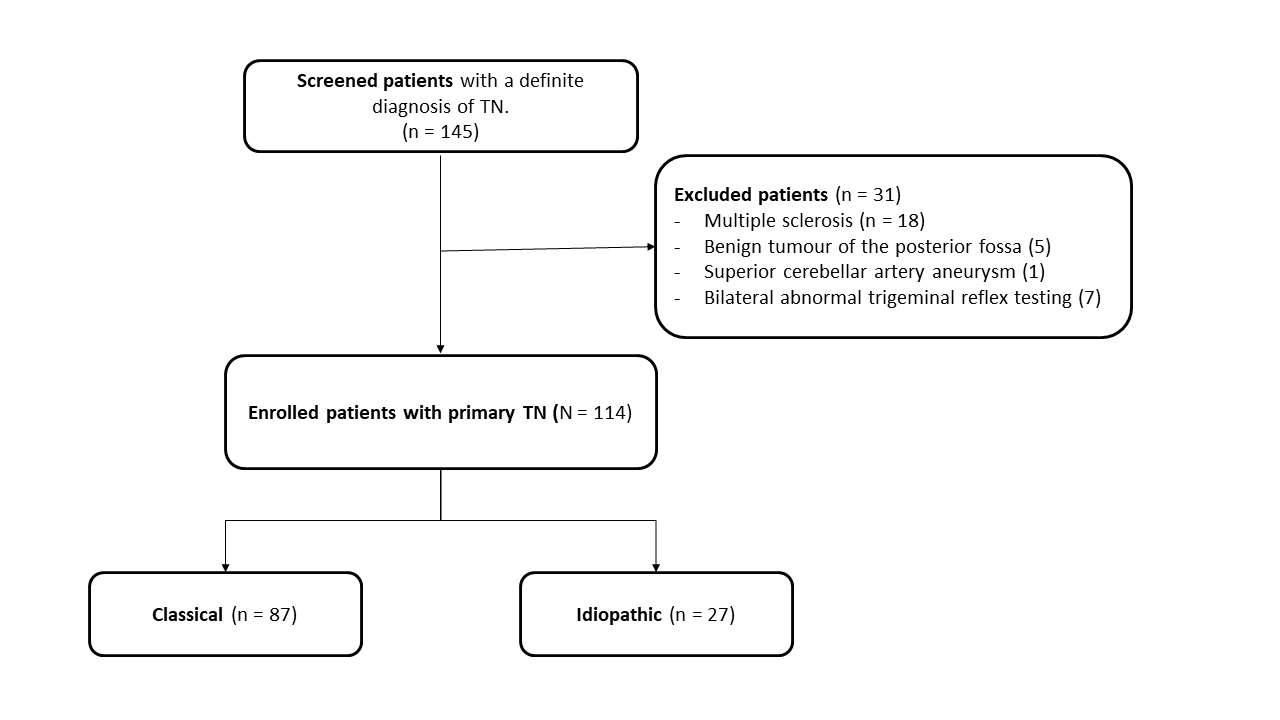

Supplement: Supplementary file 2 — Suppl. Figure. Flow diagram of patients’ enrollment. (PNG 32 kb) [file 10072_2023_6923_Fig5_ESM.png]

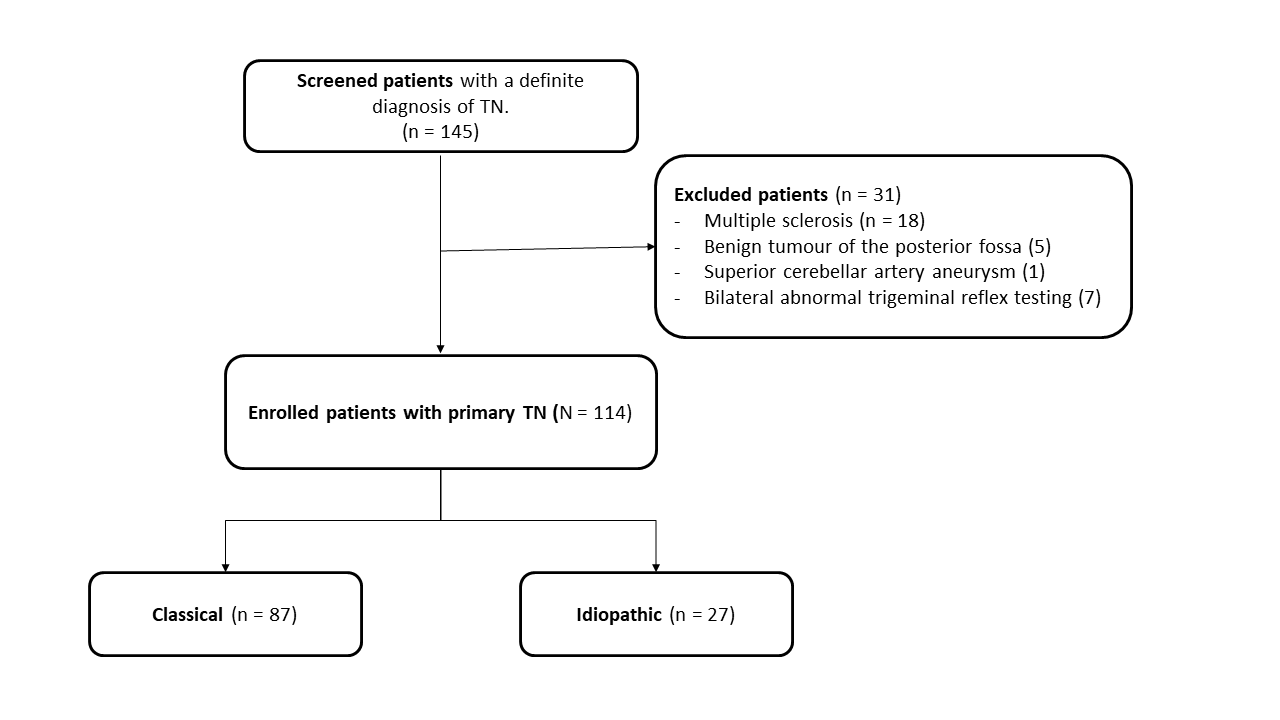

Supplement: Supplementary file 3 — High resolution image (TIF 82 kb) [file 10072_2023_6923_MOESM2_ESM.tif]
