# Supplementary material for: Sex differences in trigeminal neuralgia: a focus on radiological and clinical characteristics
Source: Neurol Sci. 2023 Jul 12;44(12):4465–72. doi: 10.1007/s10072-023-06923-5 (PMC10641090; doi:10.1007/s10072-023-06923-5)
Supplement: Supplementary file 4 — Suppl. Table. Adverse events related to carbamazepine or oxcarbazepine in 114 patients with Trigeminal Neuralgia (DOCX 13 kb) [file 10072_2023_6923_MOESM3_ESM.docx]

**Suppl. Table. Adverse events related to carbamazepine or oxcarbazepine in 114 patients with Trigeminal Neuralgia.**

| **Adverse events** | **Female** | **Male** |
| --- | --- | --- |
| Somnolence | 10 | 5 |
| Unbalance | 11 | 2 |
| Dizziness | 5 | 2 |
| Hyponatremia | 4 | 1 |
| Liver dysfunction | 2 | - |
| Anaemia | 1 | - |
